# Advantages of Sunlight

**Published:** 1881-10

**Authors:** 


### ADVANTAGES OF SUNLIGHT.

Sun-baths cost nothing, and are the most refreshing life-giving baths that one can take whether sick or well. Every housekeeper knows the necessity of giving her woollens the benefit of the sun, from time to time, and especially after a long rainy season, or a long absence of the sun. Many will think of the injury their clothes are liable to, from dampness, who will never reflect that an occasional exposure of their own bodies to the sunlight is equally necessary to their health. The sun-baths cost nothing, and that is a misfortune, for people are still deluded with the idea that those things only can be good and useful that cost money. Let it not be forgotten that three of God's most beneficent gifts to man—three things the most necessary to health—sunlight, fresh air and water, are free to all; you can have them in abundance, without money and without price, if you will. If you would enjoy good health, see to it that you are supplied with pure air to breathe all the time; that you bathe for an hour or so in the sunlight, and that you quench your thirst with water only.

A NEW material for architectural purposes, said to be entirely fireproof, is made from cotton. After being converted into a paste by chemical treatment, it is molded into the desired form and allowed to dry, when it becomes as hard as stone. It is called architectural cotton.

